# Advances in Optical and Fluorescence Imaging for Surgical Management of Thyroid Cancer

**DOI:** 10.3390/cancers18152505

**Published:** 2026-08-05

**Authors:** Blackberrie Eddins, Ting-Chun Kuo, Hyungju Kwon, Homan Kang, Maged Henary, Satoshi Kashiwagi, Robert M. Hoffman, Michael Bouvet

**Affiliations:** 1 Department of Surgery, University of California San Diego, La Jolla, CA 92037, USA; beddins@health.ucsd.edu (B.E.);; 2 VA San Diego Healthcare System, La Jolla, CA 92161, USA; 3 Department of Surgery, National Taiwan University Hospital, Taipei 100225, Taiwan; 4 Department of Surgery, Ewha Womans University College of Medicine, Seoul 07804, Republic of Korea; 5 Center for Inflammation Imaging, Department of Radiology, Massachusetts General Brigham and Harvard Medical School, Boston, MA 02115, USA; 6 Department of Chemistry, Center of Diagnostics and Therapeutics, Georgia State University, Atlanta, GA 30302, USA; 7 AntiCancer Inc., San Diego, CA 92111, USA

**Keywords:** thyroid cancer, fluorescence-guided surgery, tumor margin imaging, fluorescence imaging, tumor-targeting fluorescent probes, near-infrared fluorophores, optical imaging

## Abstract

Background/Objectives: The majority of thyroid cancer patients are diagnosed with papillary thyroid cancer. A complete R0 resection significantly lowers cancer recurrence risk for high-risk papillary thyroid cancer but can be difficult to achieve because tumor margins are not easily visualized. The purpose of the present narrative review is to describe current work and future directions in fluorescence-guided thyroid cancer surgery. Methods: PubMed and Google Scholar were used to identify 45 articles that focused on fluorescence labeling and imaging of thyroid cancer published through February 2026 using the search terms “thyroid cancer” AND (“fluorescence imaging,” OR “NIR imaging” OR “optical imaging”) OR “fluorescence guided thyroidectomy”. Standalone abstracts and publications not available in English were excluded. Results: 17 fluorescent probes were shown to visualize tumor margins and micrometastases, 3 probes were able to visualize nerves, and 2 probes targeted lymph nodes. Most studies were done using animal models. Two probes (EMI-137, which targets tumors, and bevonescein, which targets nerves) were tested in clinical trials with good safety profiles. Conclusions: Several tumor-targeted fluorophores and optical imaging strategies have shown promise for thyroid cancer localization, margin assessment, lymph node evaluation, and nerve visualization; however, most remain preclinical, and clinical utility will require standardized dosing, imaging thresholds, safety assessment, and outcome-based validation with multicenter clinical trials.

## Simple Summary

Post-operative outcomes for high-risk papillary, anaplastic, and medullary thyroid cancer patients often depend on a complete R0 resection and nerve preservation. However, R0 resection may not be necessary for low-risk papillary thyroid cancer patients. R0 resection can be difficult to achieve because tumor margins are not completely visible under traditional bright light. Multiple groups have investigated whether fluorescence imaging could enhance the precision of thyroid cancer surgery through improved visualization of thyroid tumors, cervical nerves, and lymph nodes. The purpose of the present narrative review is to describe tumor-specific fluorescent probes and optical imaging techniques under investigation for labeling thyroid cancer margins, nerves, and lymph nodes. Proposed benefits of fluorescence-guided thyroidectomy include decreasing post-operative morbidity and lowering thyroid cancer recurrence rates. However, the majority of the fluorescent probes and imaging techniques that have been investigated remain pre-clinical, and larger clinical studies are needed to determine whether there is a benefit in surgical outcomes.

## 1. Introduction

Thyroid cancer incidence dramatically increased in the early 2000s and has since plateaued, with an estimated incidence of 45,240 cases annually [1,2]. The primary treatment of thyroid cancer is surgical resection and post-operative radioiodine therapy if indicated. Although generally well-tolerated, total thyroidectomy has higher complication rates than lobectomy even with experienced surgeons and there is weak evidence to perform prophylactic central neck dissections [3,4,5]. The increased rate of thyroid cancer diagnosis has led to more total thyroidectomies and lobectomies for concerning thyroid nodules. A high number of these nodules are benign or indeterminate on post-operative pathologic analysis, which has led to surgical practice becoming more conservative with the goal of decreasing the risk of unnecessary post-operative morbidity [6]. Although surgeons are being more conservative, R0 resections with no microscopic disease remain important in the management of high-risk papillary thyroid cancer (PTC). PTC is the most prevalent thyroid cancer and accounts for 90% of cases [7]. With a complete R0 resection, the PTC recurrence rate is 14% for low-risk patients [8]. Intermediate-risk patients are more likely to have an R1 resection with microscopic residual tumor and have a recurrence rate of 43% post-operatively, and high-risk patients are more likely to have R2 resections with macroscopic residual disease and have a post-operative recurrence rate of 86%. R1 resections are often due to the inability of the surgeon to visualize tumor margins completely, while R2 resections are performed when tumors invade critical structures and cannot be safely resected. Currently, surgeons determine the extent of thyroid surgery based on tumor size, histology, and whether a patient has clinically affected lymph nodes or distant metastases. Tumor-specific fluorescent probes and optical imaging techniques are now being investigated to visualize tumor margins in thyroid cancer resection.

There are many near-infrared (NIR) fluorophores that have been studied for a variety of applications in molecular imaging and basic science research. Many of the fluorophores, like indocyanine green (ICG), are non-specific and bind to human plasma proteins. However, the fluorescent dyes can be conjugated to more specific antibodies and peptides for molecular targeting. Each probe’s source of fluorescence originates from free electrons, which can be excited from their ground state, leading to energy emission in the form of photons [9]. NIR fluorescence imaging systems typically use light sources matched to the excitation spectrum of the fluorophore, most commonly within the NIR-I window (650–900 nm), while recently developed platforms extend into the NIR-II range (1000–1700 nm) and require detectors optimized for the corresponding emission signal. Systems can be optimized for different fluorescence spectra and surgical cases, so one fluorophore like ICG may be used with a variety of imaging systems, including the Da Vinci Firefly (Intuitive Surgical Inc., Sunnyvale, CA, USA) for robotic surgery and the Stryker SPY-PHI (Stryker, Portage, MI, USA) or the Fluobeam (Fluoptics, Grenoble, France) handheld devices for open surgery. Fluorescence-guided surgery (FGS) has grown in popularity to improve tumor localization in gastric, colon, pancreatic, and breast cancer because it can be used to identify tumor margins, guide lymphadenectomy, and perform intra-operative angiography [10]. In thyroid and parathyroid surgery, near-infrared autofluorescence (NIRAF) imaging and ICG angiography have been extensively studied with multiple case reports, prospective studies, and systematic reviews on parathyroid gland identification and perfusion assessment [11,12,13]. There is evidence that FGS in thyroid and parathyroid surgery reduces post-operative day 1 hypocalcemia and may reduce the rate of permanent hypocalcemia after total thyroidectomy [14].

Intraoperative tumor labeling has the potential to lower the rate of total thyroidectomies and to decrease the extent of dissection, while also decreasing the rate of cancer recurrence. The purpose of the present study is to review the fluorescent probes and optical imaging techniques currently in use or in development to label thyroid cancers. The present narrative review expands on prior work by summarizing the fluorescent probes in comprehensive tables, including additional NIR probes, and highlighting future research needs. Fluorophores and optical imaging techniques are categorized by their role in imaging thyroid cancer, nerves, or lymph nodes, and whether they are in the preclinical or clinical phase of investigation.

## 2. Materials and Methods

**Figure 1:**
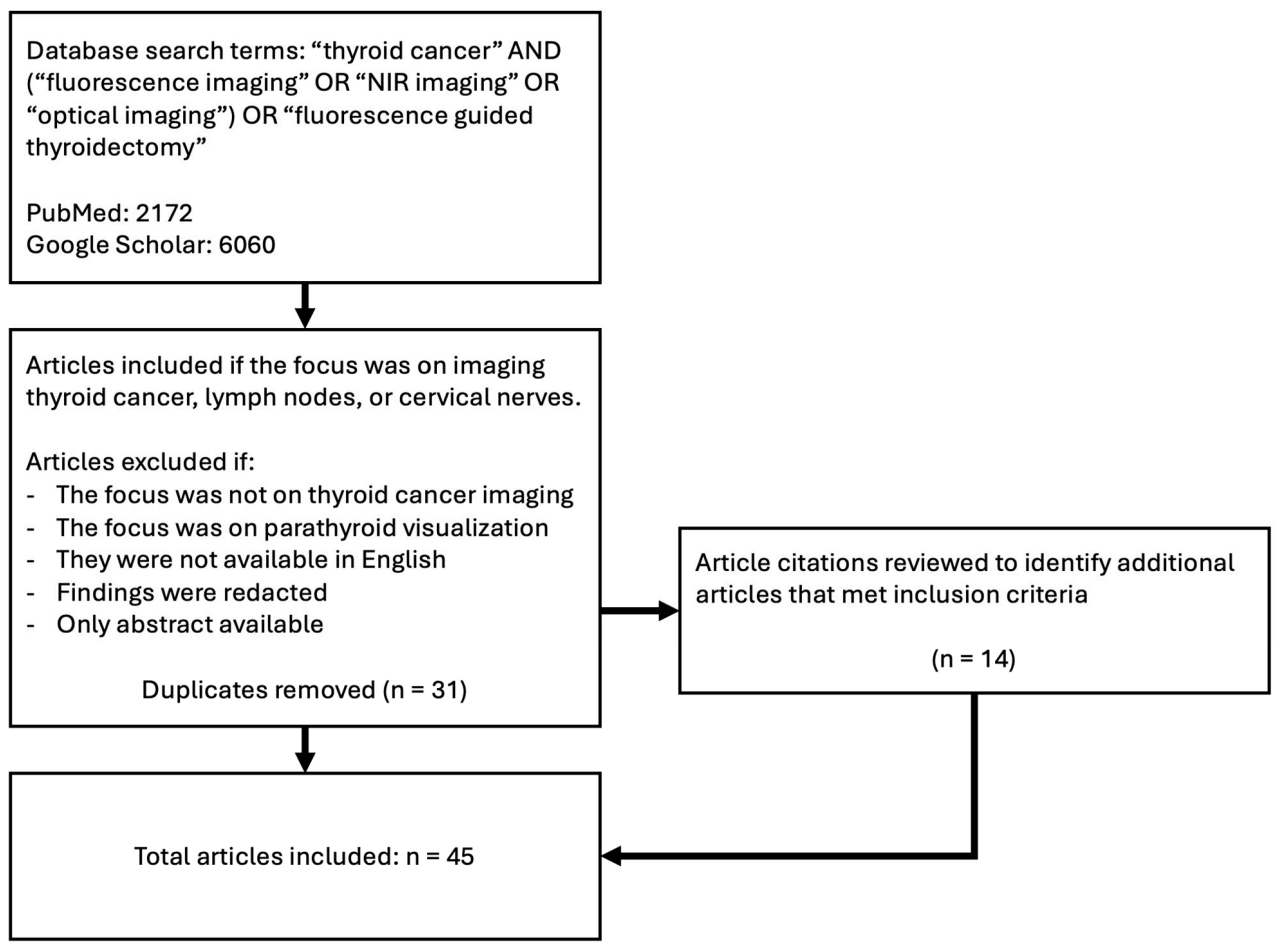
Flow diagram demonstrating how articles were searched, screened, and considered for inclusion in the present narrative review. Arrows demonstrate the order in which articles were reviewed.

The present narrative review employed the databases PubMed and Google Scholar to search for publications on the use of fluorescence imaging techniques in thyroid cancer published from January 2000 through February 2026, with the last search updated on 18 May 2026 (Figure 1). Papers were included if fluorescence imaging was used to localize thyroid cancer, lymph nodes, or nerves in the head and neck. In vitro models, animal models, and clinical use in humans were all considered. Articles were excluded if the focus of the paper was not on thyroid cancer or if the focus was on parathyroid visualization, or if only an abstract was published, or if the findings were redacted, or if publications were not available in English, or the paper was not available through Open Access or the University of California, San Diego library. The search terms “thyroid cancer” AND (“fluorescence imaging” OR “NIR imaging” OR “optical imaging”) OR “fluorescence guided thyroidectomy” were used. Abstracts were screened and categorized (B.E.) by whether the focus was tumor, lymph node, or nerve visualization, whether fluorescence imaging was performed pre- or intraoperatively, and if the probe or imaging method was tested in animal models or humans. The vast majority of articles described the use of ICG angiography or NIRAF imaging to visualize and preserve the parathyroid glands and these articles were excluded. In total, 23 articles fit the inclusion criteria from PubMed. Eight additional articles were found using Google Scholar. Article citations were reviewed to identify 14 additional papers that met the inclusion criteria for a total of 45 papers. The complete manuscripts for the 45 articles were reviewed to determine the probe or optical imaging method used, the optimal absorbance and emission wavelength (if reported), the time between probe administration and imaging (if reported), and the sensitivity and specificity (if reported). Articles were considered to have stronger evidence if they were tested in patients or ex vivo human tissue.

## 3. Results

### 3.1. Pre-Operative Tumor Localization

Neck ultrasound (US) is the gold standard in pre-operative imaging of thyroid cancer and is quoted as having a sensitivity of 88% and specificity of 86% [15]. Patients with a suspicious thyroid mass or nodule seen with US undergo fine needle aspiration (FNA) to further stratify the risk of malignancy. Some surgeons obtain computed tomography (CT) imaging prior to operating for large tumors or concern for extrathyroidal tumor invasion not captured by the US [16]. While CT is effective for selective cases, it is not recommended for routine thyroid cancer screening because the radiation exposure increases cancer risk.

FNA cytopathology malignancy risk is classified using the Bethesda System. Bethesda IV and V nodules are recommended to proceed with surgical resection; Bethesda III nodules have atypia of unclear significance; and Bethesda I and II nodules are non-diagnostic and benign, respectively [17]. Of patients with thyroid nodules that undergo surgery, it is estimated that 81% of Bethesda III nodules, 76% of Bethesda IV nodules, and 21% of Bethesda V nodules had benign final pathology [6]. Given the large number of patients who undergo unnecessary surgery as the detection rate of thyroid nodules increases, multiple groups have investigated how to improve the diagnostic accuracy of pre-operative imaging with probes that could be used for intraoperative guidance as well (Table 1).

Gold nanoclusters (AuNCs) have been investigated by many groups for their applications in imaging and therapeutics. AuNCs are fluorophores that absorb light in the NIR range, and their emission wavelength can vary from 405 nm to 1700 nm depending on the capping ligand used in their synthesis [35]. Chen et al. synthesized iodinated gold nanoclusters (AuNCs@BSA-I) with bovine serum albumin (BSA) and chloramine-T to detect thyroid tumors in orthotopic mouse models with CT and fluorescence imaging [18]. The normal thyroid gland and thyroid tumors had different patterns of fluorescence over time after injection with AuNCs@BSA-I, which could be used to improve the diagnostic accuracy of CTs. The probe had higher and more persistent fluorescence in normal thyroid tissue, which could make identifying metastases more difficult.

Another fluorescent probe tested in mouse models of thyroid disease is Mito-Cy-SEC. Mito-Sy-SEC targets selenocysteine, which is the active site of the enzymes thioredoxin reductase and iodothyronine deiodinase, making it a potential biomarker for thyroid disease. Luo et al. found that Mito-Sy-SEC fluorescence varies with selenocysteine expression and can be used to image both tumors and benign thyroid pathology [19]. The pattern of fluorescence was unique between benign and malignant thyroid disease.

**Figure 2:**
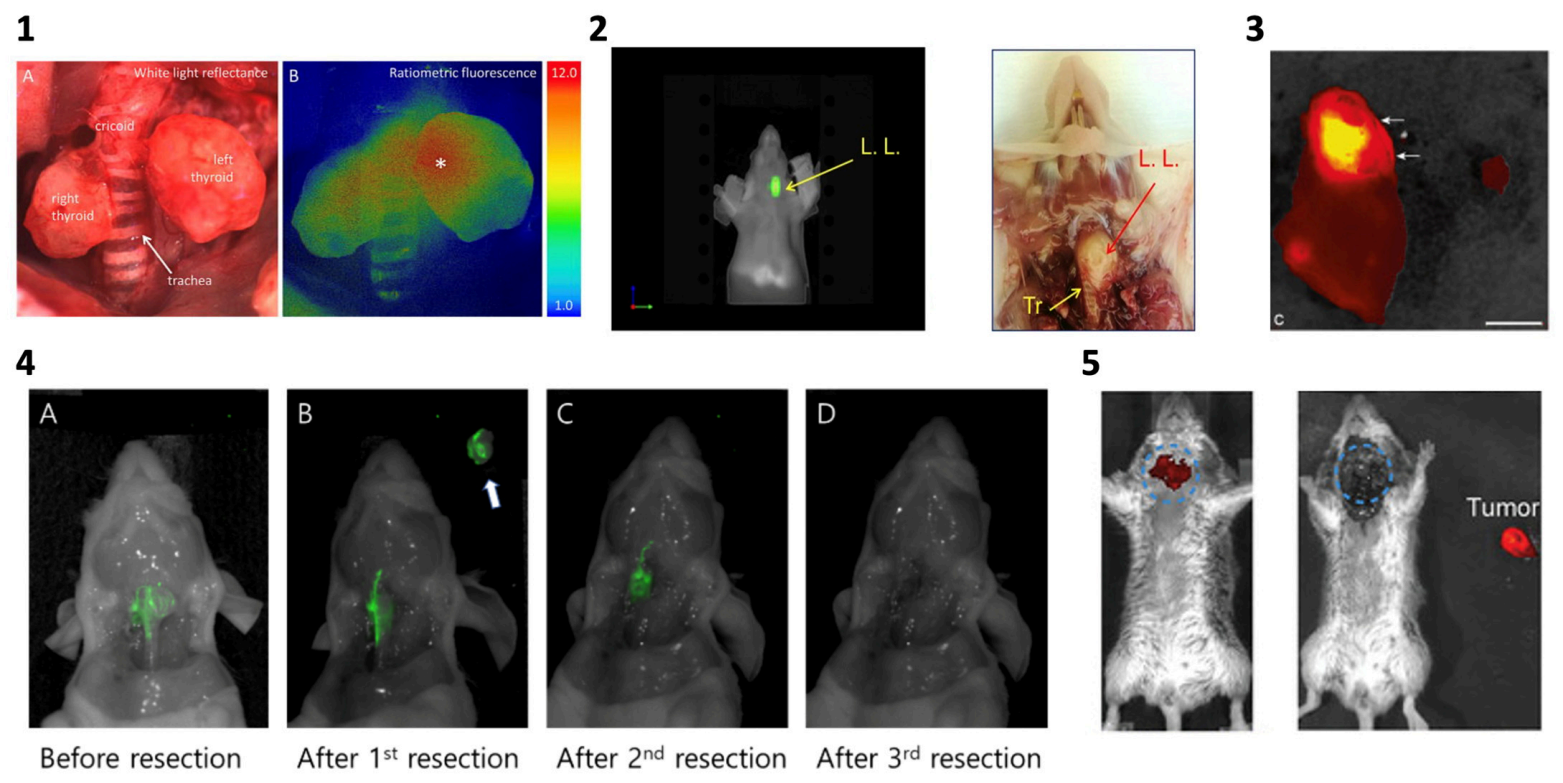
Fluorescence imaging of thyroid cancer in orthotopic mouse models (**1**,**2**,**3**,**4**) and in a patient after thyroidectomy (**5**). (**1**) A. white light image of a transgenic BRAF^V600E^ mouse thyroid gland completely replaced by tumor, B. radiometric fluorescence image of thyroid tumor labeled with RACPPgr1, asterisk (*) denotes maximal contrast ratio of 11.69 in the central area of the left thyroid lobe; from [23] with permission from John Wiley and Sons. Ratiometric scale bar shows low fluorescence ratio (blue) up to high fluorescence ratio (red). (**2**) Left: fluorescence imaging of orthotopic mouse model performed 48 h after injection of Cy5.5-labeled aGal3-F(ab′)2; fluorescence signal is shown on a scale from green (low fluorescence) to red (high fluorescence). Right: white light image of orthotopic thyroid tumor at necropsy (Tr = trachea; L. L. = left lobe); from [21] originally published by © SNMMI. (**3**) Representative IVIS Spectrum images of a patient’s resected thyroid lobe after treatment with EMI-137 (0.13 mg/kg dosage) demonstrating multifocal PTC not seen on pre-operative US. The region between the white arrows has higher fluorescence signal outside of the primary tumor, suggesting tumor deposition. Yellow represents high fluorescence signal and dark red represents low fluorescence signal, 10 mm scale bar; from [32] used under Creative Commons CC BY license. (**4**) Serial removal of margins in a K1 thyroid cancer orthotopic mouse model after spray with PH10, green represents fluorescence signal. A. before tumor resection; B. after first resection, white arrow denotes resected tumor; C. after second resection; D. after third resection, demonstrating complete tumor removal with no residual fluorescence signal; from [29] used under Creative Commons CC BY license. (**5**) Fluorescence-guided resection of orthotopic mouse model of ATC using IRDye 800CW-ALT-836, light red represents high fluorescence signal. Blue dashed circle represents tumor bed; from [24] used under Creative Commons CC BY license.

Galectin-1 expression has been shown to have a 97% specificity in distinguishing benign from malignant thyroid pathology [36]. Fanfone et al. investigated galectin-1 targeting peptides coupled to the NIR dye CF770 [20,34]. Subcutaneous mouse models of PTC were imaged non-invasively and investigators were able to distinguish between benign and malignant thyroid nodules using P7-CF770 with a sensitivity of 75% and specificity of 100%. Another galectin, galectin-3, has also been investigated for both FNA molecular testing and diagnostic imaging [21,37]. De Rose et al. used anti-galectin-3 labeled with ^89^Zr to image orthotopic mouse models of thyroid cancer and were able to distinguish benign from malignant tissue with positron emission tomography (PET)/CT and fluorescence imaging using Cy5.5 labeled aGal3-F(ab′)2 (Figure 2).

### 3.2. Intraoperative Tumor Localization

#### 3.2.1. Murine Models of Thyroid Cancer

Multiple groups have investigated the use of novel fluorescence imaging probes for intraoperative imaging of thyroid cancer. Microscopically clear surgical margins are important for PTC. Radioactive iodine can be used to ablate remnant thyroid tumor after R1 resection. Differentiated thyroid cancers have a 20% risk of local recurrence. In total, 25–50% of patients with local recurrence and many patients with distant metastasis become refractory to radioiodine therapy [38,39].

Zhang et al. developed a nitroreductase (NTR) fluorescent probe, Ox-NTR, for fluorescent imaging of PTC in subcutaneous mouse models [22]. Under hypoxic conditions, the Ox-NTR nitro group is reduced to an amine and the compound becomes the fluorophore oxazine 1. The authors proposed that the hypoxic environment of thyroid cancer leads to higher production of NTR, leading to a higher fluorescence signal after intratumoral injection of Ox-NTR. Although there was a high tumor signal with low background signal, Ox-NTR still needs to be tested in orthotopic mouse models of thyroid cancer to determine how the tumor-to-thyroid signal is impacted.

Orosco et al. used molecularly targeted radiometric activatable cell-penetrating peptides (RACPPs) in transgenic BRAF^V600E^ mice to label thyroid cancer [23]. BRAF mutations are associated with decreased iodine avidity in the thyroid and may impact clinical outcomes [40]. Fluorescence imaging with RACPPs was able to visualize two residual tumor foci after thyroidectomy. However, there were multiple false positives when imaging the tumor bed and three mice had positive margins when histopathology was analyzed. RACPPs were able to detect tumors as small as 0.8–1.2 mm, but further research is needed if the goal is to resect all micrometastases during the index operation.

Another targeted probe, IRDye800CW-ALT-836, capitalizes on tissue factor-specific monoclonal antibodies to target anaplastic thyroid cancer (ATC) tumors [24]. Tissue factor is involved in hemostasis and inflammation and tissue factor pathway inhibitor 2 inactivation has also been investigated for its potential role in thyroid cancer progression [41,42]. IRDye800CW-ALT836 was used for intra-operative guidance using a handheld Fluobeam device. Post-operative imaging showed no fluorescence signal in the tumor bed and there was minimal fluorescence signal in other organs on biodistribution. The same group also used pertuzumab conjugated to IRDye800CW for fluorescence imaging and ^89^Zr-Df-pertuzumab for PET imaging of orthotopic anaplastic thyroid cancer mouse models [25]. Pertuzumab is a HER2 monoclonal antibody currently used for the treatment of HER2-positive breast cancer [43]. HER2 is overexpressed by 44% of follicular thyroid cancers, 18% of PTC’s, and up to 16% of ATC’s [44,45]. Thyroid tumors in nude mouse models of ATC could be targeted with both IRDye800CW-pertuzumab and ^89^Zr-Df-pertuzumab using Cerenkov luminescence imaging (CLI). CLI is an optical imaging method based on detecting photons emitted by radiotracers and can be done using bioluminescence imaging systems such as the IVIS Spectrum (Revvity, Waltham, MA) or the Pearl Trilogy imaging system (LI-COR Biosciences, Lincoln, NE), which are broadly utilized in cancer and biology research [46]. Wei et al. hypothesized that ^89^Zr-Df-pertuzumab could be used for both pre-operative imaging and FGS using CLI with optimized tracer dosing and image resolution.

Rossfeld et al. also used IRDye800 conjugated to a tumor-associated probe, Compound-17, to image medullary thyroid cancer in orthotopic xenograft mouse models [26]. Their group noted that there was some background signal; however, the tumors were able to be distinguished from surrounding structures. The kidneys had the highest fluorescence signal, which was attributed to renal excretion of the dye. Similar to Wei et al., they were able to visualize subcutaneous tumors with the Fluobeam handheld device. Compound-17 is a derivative of the phage-derived peptide HN-1, which was previously used to target head and neck squamous cell carcinomas [47]. The mechanism of Compound-17 is not known; however, the authors found that the fluorescence co-localized with mitochondria in the tumor cells.

Liu et al. developed a near-infrared fluorescent polymer for delivery of siRNA to ATC tumors and were able to visualize and reduce ATC lung micrometastases in orthotopic mouse models [27]. Their group used RNAi therapy targeting BRAF, which is mutated in up to 40% of ATC patients [48]. Their probe was able to visualize small tumors and has potential for non-invasive sentinel lymph node mapping.

ICG has been used for fluorescence angiography of the parathyroid glands and for sentinel lymph node mapping but washes out quickly and is not an ideal agent for primary thyroid tumor labeling as it is not tumor specific [49]. Zhang et al. coupled ICG with ^131^I and human serum albumin (HSA) to image subcutaneous models of ATC through intratumoral injection of the dye [28]. Their probe remained within the tumor for 6–8 days, which is longer than the ICG retention time. They also performed photothermal therapy with ICG-HSA, which inhibited tumor growth but did not lead to complete tumor regression. They proposed that ICG-HSA and radioactive iodine together may be synergistic with other cancer therapies to improve response in aggressive ATCs.

The sprayable, pH-sensitive fluorescent probe (PH10) was shown to label thyroid cancer in orthotopic mouse models [29]. PH10 is more soluble and has a higher fluorescence intensity in mildly acidic conditions, which is similar to the tumor microenvironment [30]. The primary mechanism for dye uptake is via organic anion transporter peptides. PH10 was able to visualize microscopic positive surgical margins after thyroidectomy in orthotopic mouse models. Imaging can begin just 1 min after topical application, which would allow for intra-operative dosing.

Photo-acoustic imaging (PAI) has a tissue depth of penetration up to 5 cm and is an active area of research for imaging multiple cancers [50]. One group used a probe activated by matrix metalloprotease (MMP) and PAI to image subcutaneous mouse models of follicular thyroid carcinoma (FTC). Prior to imaging, the authors performed intravenous and intratumoral injection of a peptide attached to the fluorescent molecule Alexa750 that is preferentially cleaved by MMP-2 and MMP-9 in the tumor and thereby activates the fluorescent probe [31]. Matrix metalloprotease expression differs between benign and malignant thyroid nodules and has been investigated as a possible biomarker for FTCs [51]. With an optimized probe, PAI could be used for diagnosis in conjunction with pre-operative US and in the operating room when identifying tumor margins.

#### 3.2.2. Clinical Trials

The U.S. Food and Drug Administration (FDA) has cleared several fluorescent or optical contrast agents for use in surgical or procedural imaging, including ICG, 5-Aminolevulinic acid (5-ALA), methylene blue, Cysview (hexaminolevulinate), fluorescein sodium, pafalocianine (Cytalux), and pegulicianine (Lumisight) [52]. These agents differ substantially in their excitation and emission ranges, targeting mechanisms, and indications for clinical use. There are two dyes capable of labeling thyroid cancers that have been tested in patients: EMI-137 (clinical trial NCT03470259) and gGlu-HMRG. There is also one novel optical imaging technique, optical coherence imaging, which has been tested in ex vivo patient tumors.

A multicenter phase 1 clinical trial in the UK used EMI-137 to detect multifocal PTC after hemithyroidectomy or total thyroidectomy in 14 patients [32]. They were able to re-stage 80% of the patients presumed to have unifocal PTC based on pre-operative US to multifocal PTC. EMI-137 targets MET, which is a tyrosine-kinase receptor overexpressed by an estimated 95% of PTCs [53]. EMI-137 is administered 1–3 h prior to imaging, which can be done on the day of surgery. Ongoing clinical investigations will determine if the dye has an impact on clinical outcomes.

The sprayable fluorescence imaging probe gGlu-HMRG was used to image ex vivo human thyroid tissue. gGlu-HMRG targets γ-glutamyltranspeptidase (GGT), a cell surface enzyme which is overexpressed in breast, ovarian, lung, colon, and head and neck cancers [54]. The sprayable dye was able to differentiate benign thyroid pathology including thyroiditis and Graves’ disease from PTC with an estimated sensitivity and specificity of over 95%. Hino et al. hypothesized that gGlu-HMRG could eventually be used in place of frozen section, which is currently used when margins are not certain [33]. A sprayable dye with rapid results would decrease the time to diagnosis and interpreting the results does not require a trained pathologist or lab technician.

Advanced NIR imaging techniques can be used to image thyroid cancers without needing a fluorophore. Optical coherence tomography (OCT) uses low-energy NIR light to enhance tissue imaging and can determine benign from malignant thyroid nodules [55,56,57]. Rubinstein et al. showed that surgeons can use OCT in vivo to distinguish benign thyroid, thyroid nodules, and thyroid cancer with high accuracy, but had lower success with differentiation between normal parathyroid, parathyroid adenomas, involved lymph nodes, and fat [58]. OCT can also be used to detect microscopic extrathyroidal tumor extension in ex vivo thyroidectomy specimens of patients with PTC with an approximate sensitivity of 81% and specificity of 86% [59]. A major disadvantage of OCT is the imaging depth of approximately 2 mm, hindering its ability to screen deeper nodules pre-operatively. Diffuse optical tomography (DOT) has a deeper imaging depth than OCT of 1 to 1.5 cm. It uses a photodetector to capture light scattered through tissue to generate a 3-dimensional image. Mimura et al. were able to image the thyroid with this technique but it had multiple limitations, including a refractive index mismatch between the tissue and the trachea [60]. Further work is needed before DOT can be used to reliably detect thyroid cancer.

### 3.3. Nerve Imaging

A rare but significant complication of thyroidectomy is recurrent laryngeal nerve (RLN) injury [61]. Intraoperative neuromonitoring is widely used but improved nerve identification remains an important area of research [62]. One method of visualizing nerves is through near-ultraviolet imaging, which involves filtering ultraviolet nerve autofluorescence through a handheld device (Dendrite, Gera, Germany). This can be used alone or in tandem with artificial intelligence to visualize nerves and their predicted path to avoid RLN injury and was found to have high sensitivity and specificity [63,64,65,66]. The benefit of this technology is that it does not require a probe to be administered to the patient, which may have systemic toxicity.

**Figure 3:**
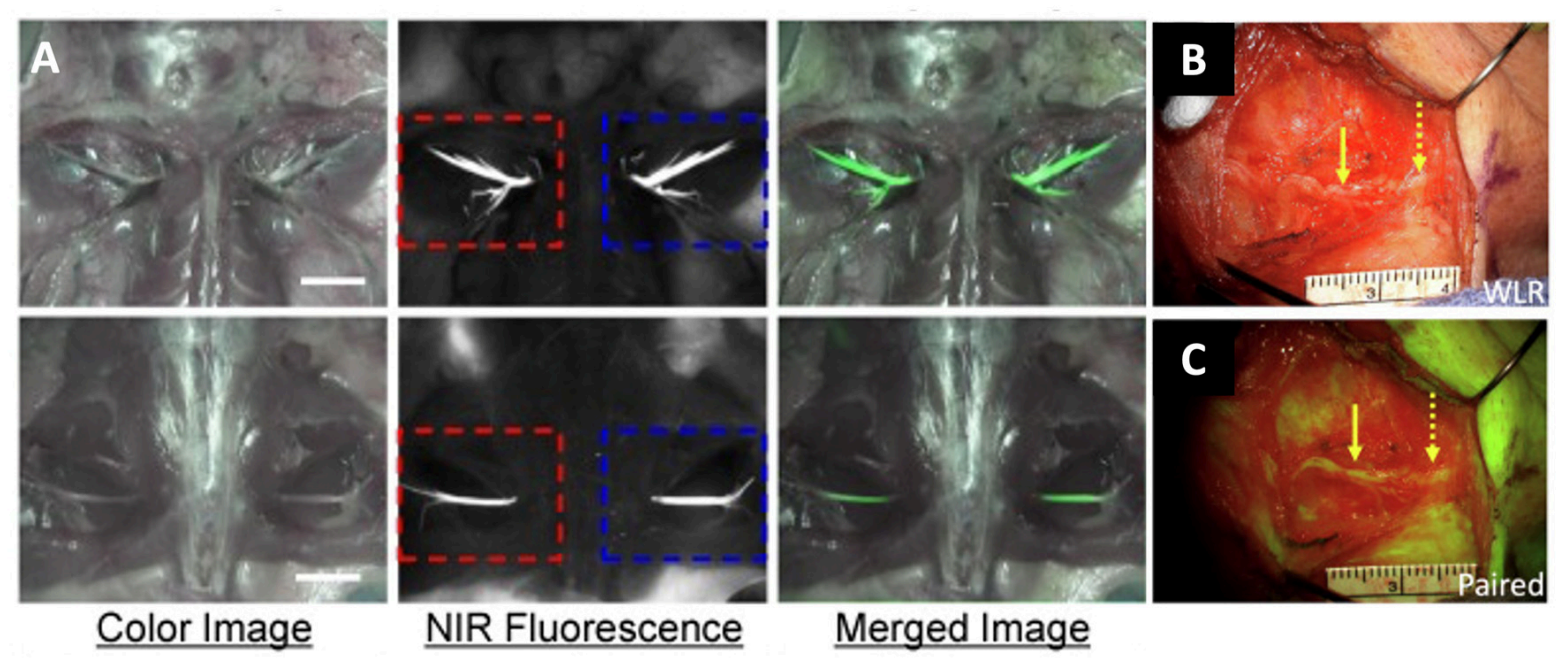
In vivo nerve-specific targeting. (**A**) Identification of the brachial plexus in rats 4 h after intravenous injection of Oxazine 4 (1 µmol dosage). The white-light (color) image, NIR fluorescence image, and a merged image are shown; the red rectangle denotes the right brachial plexus, and the blue rectangle denotes the left brachial plexus; scale bars = 1 cm; from [67] used under Creative Commons CC BY-NC license. (**B**,**C**) Paired intraoperative nerve images in patients after being given bevonescein (500 mg dose). (**B**) White-light image, (**C**) fluorescence image overlay with dashed yellow arrow showing a nerve not on the surgical field is more easily discernible from adjacent tissue (solid yellow arrow denotes nerve in the surgical field); from [71] used under Creative Commons CC BY-NC-ND 4.0 license.

There are two promising nerve-targeted dyes which have been studied in animal models and ex vivo human nerves (Table 2). One of these is oxazine 4, which was used to visualize the recurrent laryngeal nerve in swine and the brachial plexus and sciatic nerves in mice and rats (Figure 3) [67]. Oxazine 4 is proposed to target myelin or a closely associated molecule. Topical oxazine derivatives have also been tested in live swine and have been shown to have a brighter signal than systemic intravenous injection [68,69]. A benefit of topical dye application is decreased systemic absorption and a more favorable safety profile. Another dye tested in animal models is the peptide dye conjugate FAM-HNP41, which is derived from fluorescein and was shown to visualize autonomic nerves in mice and rats [70]. The same group used phage-display peptides labeled with fluorescein to create FAM-HNP401, which selectively binds to human nerves ex vivo.

Bevonescein is a variant of FAM-HNP401 which more selectively highlights axons and has been tested in Phase I and II clinical trials [71,72]. The phase I trial found no dose-limiting toxicities or infusion reactions. The signal-to-background ratio of the nerves visualized was significantly higher with fluorescence imaging than with white light and surgeons reported more confidence in nerve discrimination. Lee et al. used a surgical microscope for quantitative imaging in the trial [71]. The investigators also developed a wearable system that integrates with surgical loupes for ease of use in the operating room.

### 3.4. Lymph Node Dissection

Currently, central neck lymph node dissection is recommended by the American Thyroid Association for patients with differentiated thyroid tumors > 4 cm (cT3a), tumors with extra-thyroidal extension (cT3b or cT4), clinically affected central lymph nodes (cN1), or distant metastasis (cM1) [3]. Multiple groups have investigated how to target sentinel nodes with fluorescence imaging.

A prospective study including 110 patients diagnosed with papillary thyroid cancer was split into a fluorescence-guided surgery and white-light surgery group [73]. They found that slow injection of a small volume of ICG (0.05 mL, 1.25 mg/mL) into the thyroid parenchyma was ideal for imaging the lymphatics and avoiding dye spillage. There was a significant difference in the number of lymph nodes retrieved 7.0 ± 3.9 in the fluorescence-guided group and 4.8 ± 3.1 in the control group (*p* = 0.004) and no significant difference in other patient pre or post-operative factors. Kuczma et al. reviewed this and 2 other studies which investigated fluorescence imaging in central lymph node dissection for thyroid cancer [74]. Overall, there was evidence that NIRAF and ICG angiography can be used during lymph node dissection to lower the risk of post-operative hypoparathyroidism. Another group used ICG in combination with carbon nanoparticles to increase the number of lymph nodes identified [75]. In this study, surgeons injected a mixture of ICG and carbon nanoparticles into the middle lobe of the thyroid for lymphatic mapping.

In addition to labeling PTC metastases as described above, EMI-137 was used to target metastatic lymph nodes [76]. It was most useful for ruling out metastatic lymph nodes with a sensitivity of 94% and specificity of 26%.

Becker et al. used multispectral optoacoustic tomography (MSOT) to characterize reactive and metastatic cervical lymph nodes in patient-derived specimens ex vivo [77]. MSOT uses the photoacoustic effect of a pulsed laser causing thermoelastic expansion in tissue to image how optical absorbance varies across multiple wavelengths with an ultrasound probe. Different chromophores (water, oxygenated and deoxygenated hemoglobin, etc) appear differently as the laser wavelength is varied. The system they used (MSOT Acuity Echo research system, iThera Medical, Munich, Germany) has more recently been tested in a clinical trial where it safely imaged breast tumors and sentinel lymph nodes [78]. Dima et al. developed a handheld MSOT system which was able to image vascular networks near the thyroid at a higher resolution than traditional Doppler ultrasound [79]. PAI may play a role in determining the extent of central neck lymph node dissection and surgical planning in the future.

## 4. Discussion

In the present study, we have identified 17 fluorophores which label thyroid cancers, 3 which label nerves, and 2 which label lymph nodes. Additionally, there are 5 novel imaging techniques in various stages of development for diagnostic and intraoperative imaging in the surgical treatment of thyroid cancer. Although there are a multitude of fluorophores being studied for labeling thyroid cancers intraoperatively, the majority of these are in the pre-clinical phase. The fluorescent dyes EMI-137 and bevonescein have been safely tested in patients and are the closest to routine clinical use. Further toxicology and dosing studies will be necessary prior to using the other 15 probes in the clinic, which are expensive and time-consuming and have many pitfalls, including upscaling probe manufacturing, dye purification, and differences between animal and human drug metabolism and effects. Additionally, current intraoperative fluorescence imaging systems are optimized for the emission and absorbance spectra of ICG (780–790 nm/810–830 nm). Probes with peak emission spectra significantly higher or lower than ICG (AuNCs@BSA-I, Cy5.5-labeled aGal3-F(ab′)2, Ox-NTR, gGlu-HMRG)_would require new intraoperative fluorescence imaging systems to be developed and optimized, which would add to the expense and resources needed for clinical translation. Novel imaging techniques which are under investigation may be translated to the clinic more readily than fluorescent probes since they do not introduce toxicity to the patient and there are pre-existing laser safety ranges already documented [80]. Of these, near-ultraviolet imaging has already been used intraoperatively for nerve imaging, and MSOT can be readily translated for use in central neck lymph node imaging based on its success in breast cancer patients. However, these imaging techniques may also be prohibitively expensive to build at a large enough scale for clinical trials and may require updated designs to account for working space in the operating room, sterility, and product reusability.

In addition to the research needed to translate novel fluorophores from the pre-clinical to clinical use, there is a higher cost associated with the use of new imaging techniques in the operating room. Purchasing new devices and their associated equipment has high start-up costs, and additional charges are added for the use of any dyes, disposable parts, or covers necessary to maintain sterility. A review of current devices used in the operating room by Preziosi et al. found that the cheapest device on the market costs almost $32,000, with more complicated devices in the range of $200,000 [81]. Some imaging probes have overlapping fluorescence and absorbance spectra with ICG, which allows operators to use systems their institution may already own. Others may need more specialized detectors to optimize their signal-to-background ratio. Most of the probes described in the present study require a fluorescence imaging system, except bevonescein, which can be used with any microscope or wearable loupes.

Currently, pre-operative US and CT are the main imaging modalities available to endocrine surgeons. Thyroidectomies are typically performed under bright light with fluorescence imaging reserved for clinical trials. A major proposed benefit of labeling thyroid cancers is to obtain an R0 resection without remnant micrometastases. However, there are conflicting opinions about the importance of negative margins in well-differentiated thyroid cancers. A retrospective review including 2616 patients published in 2015 found no significant difference in local recurrence-free survival between patients with and without microscopic positive margins when adjusting for tumor extent and pathologic tumor stage [82]. This is in contrast to prior studies, which have shown incomplete tumor resection is associated with worse outcomes [83]. It is unclear whether increased use of fluorescence in thyroid cancer surgery will impact patient clinical outcomes such as mortality and disease-free survival and larger multicenter clinical trials are needed. It is possible that as more surgeons incorporate FGS into their practice, we will be able to measure the impact of a more efficient dissection. Additionally, FGS has the potential to improve the surgical confidence of less experienced surgeons who are either early in their practice or in rural settings without surgeons specialized in endocrine surgery.

A limitation of the present review is that it focused on papers that specifically investigated the use of NIR imaging during thyroidectomies. We did not review the literature to find fluorophores targeting other potential biomarkers which crossover to other cancer types like breast, colon, and other head and neck cancers. Not every paper reported the emission and fluorescence spectra of their dye, and many did not compare how their probe performs in benign diseases of the thyroid to thyroid cancer to determine sensitivity and specificity. Neither effective imaging depth nor tumor-to-background ratios were routinely reported, highlighting key areas for future research comparing the clinical utility of different fluorescent probes. In addition to the lack of standardized outcomes, there was heterogeneity in study design with both murine models and clinical trials included, which cannot be compared directly. Safety data was available for the two dyes that were investigated in clinical trials, EMI-137 and bevonescein, and was limited in the preclinical studies.

### Future Perspectives

The International Society for Fluorescence Guided Surgery (ISFGS) conducted an international Delphi survey of experts in fluorescence-guided thyroid and parathyroid surgery [84]. Their focus was the use of autofluorescence imaging and ICG for identification of the parathyroid glands. Experts agreed that allergic reactions to ICG are rare and that autofluorescence and ICG perfusion techniques are useful for identifying parathyroid glands and training residents. Although international surgical societies do not think there is enough evidence to support routine use of ICG for parathyroid gland identification, its use is expected to increase in the next decade [85]. It is only natural that FGS will continue to expand to include more targeted dissections of thyroid tumors, nerves, and lymph nodes as more fluorescent probes are cleared for clinical use.

In the future, we anticipate that more molecular targets will be identified that differentiate between thyroid tumors and benign thyroid lesions with high sensitivity and specificity. These targets will likely be used to formulate fluorescent dyes with emission spectra in the NIR-II range, which has decreased background autofluorescence, allowing for improved signal-to-noise ratios. NIR-II intraoperative imaging systems will need to be developed for FGS, and artificial intelligence (AI) algorithms will likely be trained for automated detection of micrometastatic thyroid cancer. Additionally, MSOT will likely be optimized for improved pre-operative imaging for lymph node staging and potentially adding more information to determining the pre-operative risk of cancer for indeterminate thyroid nodules.

## 5. Conclusions

There are many promising new fluorescent probes and imaging techniques for pre-operative diagnosis and intraoperative labeling of thyroid cancer. Pre-clinical studies have shown tumor-specific fluorophores can be highly sensitive in detecting tumor metastases, residual tumor margins, nerve involvement, and sentinel lymph nodes. Each dye’s mechanism for labeling thyroid cancer is different, highlighting the importance of identifying molecular biomarkers for improving FGS. More work is needed to translate these dyes to clinical applications through toxicity and dosing studies. Novel imaging modalities such as MSOT are closer to clinical translation but require testing in the sterile operating room environment and imaging technique standardization. There are some probes in clinical trials, including EMI-137 and bevonescein, which may have a positive impact on patient outcomes as their usage increases. Fluorescence imaging could allow surgeons to operate more conservatively and more accurately with the potential to improve oncologic outcomes for high-risk thyroid cancer patients without sacrificing patient quality of life. However, these proposed benefits have not yet been sufficiently demonstrated in the current literature and multicenter clinical studies are needed to determine the true impact on surgical outcomes.

## Figures and Tables

**Table 1:** There were 17 fluorophores that label thyroid cancers identified in the present narrative review of published literature through February 2026 that were used for either pre-, intra-, or post-operative imaging. Below are their names, emission and absorbance spectra, lead time prior to imaging, and sensitivity and specificity when reported. The majority of probes were tested in orthotopic mouse models; however, EMI-137 and gGlu-HMRG were tested in human subjects. A dash “-” indicates information that was not available in the cited article or its Supplementary Materials. MRI: magnetic resonance imaging, PET: positron emission tomography, ATC: anaplastic thyroid cancer, MTC: medullary thyroid cancer, PAI: photoacoustic imaging.

Study	Probe	Absorbance/Emission Wavelength	Imaging Time	Sensitivity/ Specificity	Advantages	Limitations
*Chen* et al. 2017 [18]	AuNCs@BSA-I	400–510/680 nm	1.5 h	Tumors ≥ 2 mm per image	Low toxicity	Mouse model, high thyroid fluorescence
*Luo* et al. 2020 [19]	Mito-Sy-SEC	630–730/720–840 nm	-	-	Applications in benign and malignant lesions	Pre-clinical/mouse model
*Fanfone* et al. 2020 [20]	P1-CF770	770/797 nm *	1–2 h	50%/50% per lesion	MRI-compatible	Subcutaneous mouse model, false positives
*Fanfone* et al. 2020 [20]	P7-CF770	-	1–2 h	75%/100% per lesion	MRI-compatible	Subcutaneous mouse model
*De Rose* et al. 2019 [21]	Cy5.5 labeled aGal3-F(ab′)2	-	-	-	Compatible PET tracer	Pre-clinical/mouse model
*Zhang* et al. 2024 [22]	Ox-NTR	635/683 nm	10 min–2 h	-	Very bright	Subcutaneous mouse model, intratumoral dye injection
*Orosco* et al. 2016 [23]	RACPPs	-	2 h	Tumors ≥ 1.2 mm per animal **	Determined survival after surgery	False-positives, experimental imaging technique, mouse model
*Wei* et al. 2020 [24]	IRDye800CW-ALT-836	745/800 nm	7 days	-	Targets ATC, non-invasive imaging	Pre-clinical/mouse model
*Wei* et al. 2019 [25]	^89^Zr-Df-pertuzumab	-	24 h	-	Targets ATC, PET-compatible	Mouse model, experimental imaging technique
*Wei* et al. 2019 [25]	IRDye800CW-pertuzumab	745/800 nm	3–6 days	-	Targets ATC, non-invasive imaging	Pre-clinical/mouse model
*Rossfeld* et al. 2017 [26]	Compound-17 (IRDye800–labeled HN-1 analog)	-	48 h	-	Targets MTC	Pre-clinical/mouse model
*Liu* et al. 2016 [27]	siRNA nanoparticle	710/825–850 nm	24 h	-	Targets ATC micrometastases, therapeutic	Pre-clinical/mouse model
*Zhang* et al. 2022 [28]	ICG-HSA-^131^I	808/-	1 h–7 days	-	Targets ATC, CT-compatible, therapeutic	Pre-clinical/mouse model
*Kwon* et al., *Jaiswal* et al. 2026 [29,30]	PH10	589–641/780–796 nm	1 min	-	Topical, rapid	Pre-clinical/mouse model
*Levi* et al. 2013 [31]	Alexa750-CXeeeeXPLGLAGrrrrrXK-BHQ3	700/720–800 nm	100 min	-	PAI-compatible	Subcutaneous mouse model
*Metman* et al. 2024 [32]	EMI-137	640/675 nm	1–3 h	Tumors ≥ 1.4 mm per image **	Tested in Phase I clinical trial	Outcomes research needed
*Hino* et al. 2018 [33]	gGlu-HMRG	497/523 nm	-	>95%/>95% *** per lesion	Ex vivo human tissue, compared benign pathology **	Clinical trial needed

* Absorbance and emission spectra of CF770 as reported by AAT Bioquest, Inc. [34]. ** Pathologic diagnosis was based on hematoxylin and eosin staining. *** Estimate reported by authors; measured sensitivity and specificity in the paper were 100% and 100%, respectively.

**Table 2:** There were 3 fluorophores that label nerves during thyroidectomy identified in the present review of published literature through February 2026. Below are their names, emission and absorbance spectra, lead time prior to imaging when reported, advantages, and limitations. A dash “-” indicates information that was not available in the cited article or its Supplementary Materials.

Study	Probe	Absorbance/Emission Wavelength	Imaging Time	Advantages	Limitations
*Park* et al. 2014 [67]	Oxazine 4	200–1100/350–1000 nm	1–4 h	topical application, high-contrast	pre-clinical/animal models
*Hingorani* et al. 2018 [70]	FAM-HNP41	-	2–6 h	tested in human tissue ex vivo	pre-clinical
*Lee* et al. 2025 [71]	Bevonescein	480–490/530 nm	1–2 h	Phase I and II clinical trials, low-cost peptide, simple imaging system	high background signal

## Data Availability

No new data were created or analyzed in this study. Data sharing is not applicable to this article.

## Abbreviations

The following abbreviations are used in this manuscript:

PTC	Papillary thyroid cancer
NIR	Near-infrared
ICG	Indocyanine green
FGS	Fluorescence-guided surgery
NIRAF	Near-infrared autofluorescence
US	Ultrasound
FNA	Fine needle aspiration
CT	Computed tomography
MRI	Magnetic resonance imaging
PET	Positron emission tomography
ATC	Anaplastic thyroid cancer
MTC	Medullary thyroid cancer
PAI	Photoacoustic imaging
AuNC	Gold nanocluster
BSA	Bovine serum albumin
NTR	Nitroreductase
RACPPs	Radiometric activatable cell-penetrating peptides
CLI	Cerenkov luminescence imaging
HSA	Human serum albumin
MMP	Matrix metalloprotease
FTC	Follicular thyroid carcinoma
FDA	Food and Drug Administration
5-ALA	5-Aminolevulinic acid
GGT	γ-Glutamyltranspeptidase
OCT	Optical coherence tomography
DOT	Diffuse optical tomography
RLN	Recurrent laryngeal nerve
MSOT	Multispectral optoacoustic tomography
ISFGS	International Society for Fluorescence Guided Surgery
AI	Artificial intelligence
